# Ionic liquids strongly affect the interaction of bacteria with magnesium oxide and silica nanoparticles†

**DOI:** 10.1039/c9ra05110d

**Published:** 2019-09-12

**Authors:** Andrzej Borkowski, Marcin Syczewski, Anna Czarnecka-Skwarek

**Affiliations:** Faculty of Geology, University of Warsaw Żwirki i Wigury 93 02-089 Warsaw Poland a.borkowski@uw.edu.pl; Faculty of Geology, Geophysics and Environmental Protection, AGH University of Science and Technology Al. Mickiewicza 30 30-059 Kraków Poland

## Abstract

Quaternary ammonium theophylline-based ionic liquids and imidazolium-based ionic liquids, magnesium oxide and silica nanoparticles were used in order to investigate the interaction with Gram negative *Escherichia coli* and Gram positive *Bacillus cereus*. The changes of bacterial sensitivity to both nanoparticles (NPs) and ionic liquids (ILs) were examined. In order to assess the impact of ILs on the interaction of nanoparticles with bacteria, respirometric analysis, activity of dehydrogenases, peroxidase analyses as well as scanning and fluorescence microscopy examinations were conducted. The interactions of ILs with nanoparticles based on adsorption and sedimentation tests were also investigated in order to assess how the ILs affect the agglomeration of NPs. It was assumed, as the main hypothesis of the present studies, that the differences in sensitivity of bacteria to combined ILs and NPs can be observed, even if the concentration of both compounds are below the minimum inhibitory concentration (MIC). The results indicated that ILs strongly affected the sensitivity of bacteria to nanoparticles however, the changes of sensitivity depended on the surface characteristics of the nanoparticles. The presence of ILs at non-lethal concentrations caused an increase of bacterial sensitivity to MgO nanoparticles. Notably, the sensitivity of Gram positive bacteria increased significantly when ILs were present. This was an important observation because the toxicity of nanoparticles toward Gram positive bacteria is usually lower than their toxicity toward Gram negative bacteria. Using silica nanoparticles, the presence of ionic liquids caused the adsorption of bacteria onto the surface of nanoparticle agglomerates. In conclusion, two opposing effects have been observed. On the one hand, the toxicity of MgO NPs in the presence of ILs has increased. On the other hand, the presence of silica nanoparticles caused a decreased sensitivity of both types of bacteria toward ILs. Our studies indicate potentially useful processes in many environmantal protection technologies like water treatment where flocculation and disinfection are extremely needed.

## Abstract 2

The antibacterial properties of nanoparticles can be strongly affected by interactions with ionic liquids.

## Graphical abstract

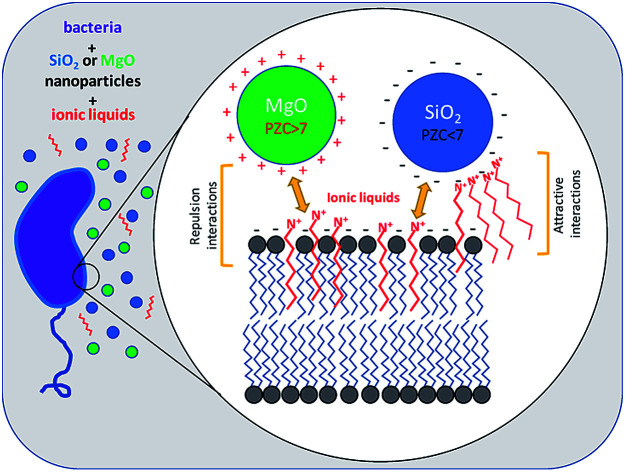

## 1. Introduction

Ionic liquids (ILs) are regarded as promising agents in the chemical industry replacing toxic organic solvents. It is known as “green chemistry”, however, these amphiphilic compounds seem not to be inert for living organisms. Similarly, their chemical character suggests the possibility of surface interaction with mineral phases which can lead to physical phenomena like agglomeration and flocculation of small particles. Hence the interaction between ILs (and other amphiphiles) and nanoparticles seems to be very important from the technological point of view. Due to the very diverse surface chemistry of the nanoparticles, they were very often functionalized in order to obtain desired properties usable in both technological processes, diagnosis and killing the pathogenic bacteria.^1–6^ Besides the studies involving synthesis and applications of both ILs and nanomaterials, their interactions with living cells are also crucial. ILs can strongly interact with both bacterial and eukaryotic cells to cause lethal or cytostatic effects.^7–16^ Similarly, nanomaterials can also exhibit toxic properties.^17–22^ However, studies concerning the toxicity of ILs and nanomaterials are usually conducted separately. The surface properties of nanomaterials can be affected by amphiphilic compounds, which can in turn cause changes of sensitivities toward microorganisms. However, ILs can strongly interact with cell membranes, which are also responsible for the sensitivity to nanoparticles. It is possible that such changes of bacterial sensitivity are linked with the modification of electrokinetic potential, modification of membrane properties, or the formation of nanoparticle agglomerates in IL solutions. It was previously shown that ILs can interact with bacterial lipid monolayers, where they can modify bacterial membrane properties to change the fluidity and even net surface charge.^7,23,24^ Such interactions can cause changes of permeability and lipid peroxidation, which in turn leads to the generation of oxidative stress.^25^ The toxicity of ILs is strongly correlated with their chemical structure. Because longer alkyl substituents can more easily interact with hydrophobic regions of cell membranes, a longer aliphatic chain in the structure usually causes a more toxic effect.^7,11,23,26–28^ However, the cut-off effect can be observed and the toxicity of ILs with long aliphatic chain usually decreases due to lower solubility. Regarding nanostructures, the toxic properties may involve both the mechanical damage to membranes and the surface activity leading to the generation of reactive oxygen species (ROS),^29–32^ as well as the toxic ions releasing from nanoparticles.^33,34^ The interaction between living cells and nanomaterials is strongly affected by surface properties like zeta potential, surface charge and energy, wettability and hydroxylation degree.^35^ It should be emphasized that the lack of an outer membrane results in the Gram positive bacteria being more sensitive to ILs.^9^ In turn, nanomaterials can damage both types of bacteria, but in some cases the Gram positive bacteria is less sensitive.^21,36,37^

If the amphiphilic character of the ILs is responsible for their interaction with bacterial membranes, it is possible that the same properties can affect the interaction of nanoparticles with bacterial cells in aqueous solutions of ILs. When considering these types of interactions, the point of zero charge (PZC) of the nanoparticles and bacteria should be considered. Living bacteria have a negative net surface charge at neutral pH^38,39^ thus, depending on the electrokinetic potential of the NP surface, an attractive or repulsive effect is observed. In the same manner, similar interactions could be shown between ILs and nanoparticles (NPs). The ILs have a charge due to their ionic character, and also can contain a long alkyl chain. However, the ILs can intercalate into the cell membrane, and ILs can interact with NPs, depending on the surface charge. Therefore, the main hypothesis, that have been tested in the presented work, is: the differences in sensitivity of bacteria to combined ILs and NPs can be observed, even if the concentration of both compounds are below the minimum inhibitory concentration (MIC). Additionally, the observed phenomena should be dependent on the PZC of the NPs.

In the present study, imidazolium-based ionic liquids (ImILs) were used as an example of well-known ILs. Additionally, in preliminary studies the theophylline-based ionic liquids (TILs) were also examined in an assembly of quaternary ammonium compounds with C_8_–C_18_ aliphatic chains and anions of natural origin. Both types of studied ILs had an alkyl substitution in the cation region. In the case of NPs, the magnesium oxide NPs and silica NPs were used as an example of nanomaterials differing by their PZC, net surface charges as well as different catalytic properties and surface activity.

## 2. Materials and methods

### 2.1. ILs

The theophylline-based quaternary ammonium ionic liquids (alkyltrimethylammonium theophyllinates) and the alkylimidazolium chlorides were used in the present studies. The structure of these ILs are shown in Table S1 (ESI†). The theophylline-based ionic liquids were synthesized according to previously described protocol.^40^ The quaternary ammonium bromides with C_8_–C_18_ alkane chains were used as precursors of theophylline-based ILs. Octyltrimethylammonium bromide and decyltrimethylammonium bromide were obtained from Fluka (College Park GA, USA). Dodecyltrimethylammonium bromide, tetradecyltrimethylammonium bromide, and hexadecyltreimethylammonium bromide were obtained from Acros Organics (Geel, Belgium). Octadecyltrimethylammonium bromide was obtained from Sigma-Aldrich (St. Louis, MO, USA). Theophylline-based ILs were designated as C8T–C18T depending on the alkyl chain. The physiochemical characteristics including the critical micelle concentration (CMC), the electrokinetic potential, and the NMR spectra for the compounds under study have been previously reported in detail.^7^ The following imidazolium ionic liquids were obtained from Sigma-Aldrich: 1-hexyl-3-methylimidazolium chloride (87929-5G); 1-methyl-3-octylimidazolium chloride (95803-5G); 1-decyl-3-methylimidazolium chloride (690597-5G); and 1,3-didecyl-2-methylimidazolium chloride (433780-1G). The imidazolium ionic liquids were designated as C6Im, C8Im, C10Im, and DC10Im, respectively. The ionic liquids were stored in tightly sealed vessels under nitrogen.

The theophylline-based ILs and imidazolium ILs were used in minimum inhibitory concentration (MIC) measurements. For further experiments, only the imidazolium ILs were used as an example of well-characterized ILs reported in previous studies. The compounds used included poorly toxic hexylimidazolium chloride and strongly toxic didecylimidazolium chloride.

### 2.2. Nanoparticles

The magnesium oxide nanoparticles (nanopowder, 20 nm, 99% purity, 3315HT) were obtained from Nanostructured & Amorphous Materials (Houston, TX, USA). The silicon dioxide nanoparticles (nanopowder, 10–20 nm, 99.5% purity, 637238-50G) were purchased from Sigma-Aldrich. The nanoparticles were designated as MgO NPs and SiO_2_ NPs. The nanoparticles were heated before experiments (250 °C, for 12 h) in order to dehydration and sterilization.

The nanoparticle sample morphology was examined using a scanning transmission electron microscope (STEM; AURIGA; Carl Zeiss Microscopy, Jena. Germany) equipped with two energy-dispersive detectors (Quantax XFlash 6|30; Bruker Nano, Berlin, Germany). A small amount of suspension of MgO and SiO_2_ nanoparticles in water was placed on a copper grid coated with Kapton® film (Dupont, Hayward, CA, USA). After evaporation of the water, the samples were analysed using a 60 μm aperture and a 30 keV acceleration voltage. The working distance depended on the sample height, and was chosen to be approximately 2 mm.

The size of the MgO and SiO_2_ nanoparticles were investigated using a powder X-ray diffractometer (PANalytical X'Pert PRO MPD; Malvern Panalytical, Malvern, UK) using the Bragg–Brentano method. Registration was carried out with a CoKα lamp at 40 kV and 30 mA in the range of 4–100° 2*θ* with a step of 0.0260° 2*θ*. The examination was carried out at 25 °C, with 8 h necessary for each sample. The long analysis time was necessary to get the exact profile of reflections, on the diffraction pattern. The crystallites were calculated using the Scherrer formula.^41,42^

Subcritical N_2_ (77 K) gas adsorption measurements were conducted on a Micromeritics ASAP2020 instrument (Norcross, GA, USA). Before analysis, 0.3 g of dry sample was degassed to minimize interferences from gases and vapours in the 60 min measurement at 90 °C to reach a vacuum set point of 500 μmmHg with a rate of 1 mmHg s^−1^; then the sample was heated to 200 °C for 480 min. After the degas procedure, the sample tube was placed on the analysis port of the instrument where the isotherms data were collected. At first, free space was measured by using helium before measuring the adsorption isotherm. The saturation vapour pressure, *P*_0_, was measured at intervals during analysis, and the analysis bath temperatures were calculated from these values. Measurements of the SiO_2_ sample were performed by the low pressure incremental dose mode with dose amounts of 5 mL g^−1^ STP. The standard procedure of the first fixed dosing applied to the mesoporous materials were used for the MgO sample. The quantity of adsorbed gas on the solid surface was measured in the range 0.000077–0.99 *P*/*P*_0_. The Brunauer–Emmett–Teller surface area was determined in the range of 0.05–0.3 *P*/*P*_0_. In this range of values, mono- multilayer adsorption onto the pore walls in mesopores and macropores occurred. Micropore areas of the SiO_2_ and MgO were calculated by using the *t*-plot method. The Harkins–Jura equation was used for estimation of the statistical thickness of the adsorbed layer. Interpretation of the N_2_ isotherm profile provided information about the physisorption mechanism. The types of pores present in the adsorbent could be predicted by analysis of the hysteresis patterns.

The point of zero charge (PZC) of the investigated materials was analysed by potentiometric mass titrations.^43^

### 2.3. The sedimentation test and the adsorption of imidazolium ILs onto NPs

The interactions between the nanoparticles and imidazolium ILs were studied based on the adsorption of ILs onto the surface of NPs and the sedimentation rate measurements, which characterized the formation of NP agglomerates in the presence of ILs. The tests were performed in triplicate. First, the solution of imidazolium ILs (200 mg L^−1^) was prepared separately in deionized water. Then, 20 mg of MgO NPs or SiO_2_ NPs was placed in glass vessels (*a* = 50 mL) and the 10 mL of IL solution was added. The obtained suspensions were then gently mixed (80 rpm) for 3 h at 25 °C. After this time, the concentrations of IL before and after adsorption were measured using the calibration curve at a wavelength of 210 nm using the G10S UV-Vis spectrophotometer (Thermo Fisher Scientific, Madison, WI, USA). The adsorption was calculated according to the formula:
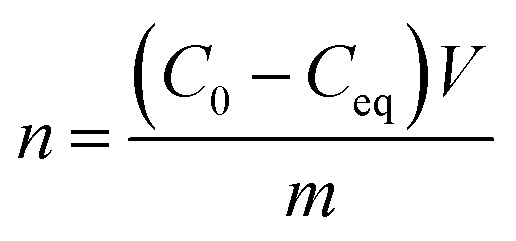
where: *n* – adsorption [mg g^−1^], *C*_0_ – initial concentration of IL [mg mL^−1^], *C*_eq_ – equilibrium concentration of IL after sorption [mg mL^−1^], *m* – weight [g].

The sedimentation test was conducted using the same samples. After the adsorption test, the suspensions of nanoparticles with IL were mixed vigorously and then, 2 mL of suspension was immediately put into the optic glass cuvette (1 mm). Next, the changes of absorbance at *λ* = 600 nm were measured for 30 min with interval 30 s. The sedimentation rate was expressed as the difference of absorbance at the initial time (0 min) and 5 min [dA t^−1^].

### 2.4. Microorganisms and media

*E. coli* K-12 was kindly provided by Prof. Jolanta Łukasiewicz from the Ludwik Hirszfeld Institute of Immunology and Experimental Therapy (Polish Academy of Sciences). The Gram positive strain, *Bacillus cereus* (ATCC 11778), was used from our collection (Geomicrobiology Laboratory, Faculty of Geology, University of Warsaw). Bacteria were cultivated in tryptic soy broth (TSB; Sigma-Aldrich), with liquid medium on agar plates containing the TSB medium.

### 2.5. Determination of the MIC

MICs were estimated using the microtiter plate method using sterile 96-well plates.^7^ First, IL liquid solutions or nanoparticle suspensions were prepared in sterile deionized water at a concentration of 20 mg mL^−1^, and 50 μL was placed in the first row of the plate. Next, 25 μL of sterile TSB medium was added to the other wells, and serial dilutions were performed. Then, 200 μL of inoculated TSB medium containing resazurin indicator (0.02 mg mL^−1^) was added to all wells. TSB medium was inoculated to the concentration of the final suspension (∼10^6^ cfu mL^−1^; about 0.5 McFarland), and the plates were incubated at 25 °C for 24 h. A colour change from blue to pink or to yellowish and an increase in turbidity were considered positive, and the lowest concentration at which there was no visible colour change was assumed to be the MIC.

In the case of MICs of ILs at ½ MIC of nanoparticles, the algorithm was the same, but the ionic liquids solutions as well as the TSB medium contained the nanoparticle suspensions at a concentration < ½ MIC. For the MgO NPs, it was the 500 mg L^−1^, for the silica NPs it was the 1000 mg L^−1^ independent of the bacteria strains. Similarly, in the case of the MIC of nanoparticles at ½ MIC of ionic liquids, the experimental setup was analogous. The nanoparticle suspensions and the TSB medium contained proper IL at a concentration of ½ MIC. However, the used IL concentrations differed dependent on the bacterial strains, which resulted from different toxic and bacterial sensitivities. We chose the ½ MIC as a concentration which does not affect significantly the microbial growth. It was important because using the ½ MIC of one xenobiotic (*e.g.* ILs), we were able to check whether the other xenobiotic (*e.g.* NPs) revealed greater or weaker toxicity in relation to bacteria exposed on these two xenobiotics simultaneously.

### 2.6. Measurements of dehydrogenase and peroxidase activities

A 10 mL aliquot from a 24 h culture of bacteria in TSB medium was mixed with the nanoparticle suspension, and imidazolium IL was added to a final concentration of ½ MIC. The cultures were incubated at 25 °C for 12 h with gentle horizontal shaking at 80 rpm. After incubation, peroxidase activity was measured as follows:^14^ 0.1 mL of culture was transferred to a 96-well microtiter plate and mixed with 0.1 mL of a 0.01 M H_2_O_2_ (Avantor Performance Materials, Poland S.A.) and 50 μL of 0.2 M pyrogallol (Sigma-Aldrich). All tests were repeated four times using four wells of the microplate. The microplate was immediately transferred to a microplate reader (Multiskan FC, Thermo Scientific, Vantaa, Finland) and the change in absorbance at 405 nm was measured over 15 min (at intervals of 15 s). The results were expressed as the change in absorbance per h (dA h^−1^) measured within the interval 1–5 min. Control cultures without studied compounds and with ILs and NPs separately were tested in parallel. The dehydrogenase activity was measured similarly; 0.1 mL of culture was transferred to a 96-well plate and mixed with 20 μL of 3% triphenyltetrazolium chloride (Avantor Performance Materials, Poland S.A.) and the change in absorbance at 405 nm was measured over 30 min (at intervals of 1 min). The results were expressed as the change in absorbance per h (dA h^−1^) measured within interval 1–30 min. The controls were the same as described above.

### 2.7. SEM analysis

The cultures used for enzymatic analyses (as described above) after incubation were examined using an AURIGA scanning electron microscope (Carl Zeiss Microscopy, GmbH). The cultures with MgO NPs, SiO_2_ NPs, and C6Im were chosen for microscopic analysis. Additionally, the cultures without studied compounds were also investigated as control samples. Each sample was fixed with glutaraldehyde solution by adding 50 μL of glutaraldehyde (50%) to 1 mL of the sample. The fixation was carried out for 10 min. The samples were then centrifuged for 2 min at 8000 × *g*. Then the residues were gradually suspended in 1 mL of 50%, 70%, and 96% ethyl alcohol solutions. Before this procedure, higher concentrations of alcohol were used; samples were centrifuged for 2 min at 8000 × *g*, and 5 μL of bacterial suspension in 96% ethanol, from each sample, was applied to a microscope slide, covered with a 20 nm layer of carbon, and left to dry. The samples were then coated with a 20 nm layer of gold. Each layer of carbon was coated by a vacuum coater (Quorum 150T ES; Quorum Technologies, Lewes, UK). Furthermore, carbon tape bridges were made to avoid excessive charge accumulation. The secondary electron-mode photos were taken using a 60 μm aperture and a 20 keV acceleration voltage. The beam intensity was 1.5 nA, and the working distance was chosen at approximately 8 mm.

### 2.8. Fluorescence microscopy

Fluorescence microscopy was conducted in order to assess the formation of bacterial aggregates in the presence of ILs and nanoparticles. For the analysis, C6Im was chosen due to its low toxicity and its high MIC value. These conditions minimalized the mistakes caused by an excess of concentration adding to the small amount of bacterial suspension before the experiment. First, the bacterial suspension in sterile 0.9% NaCl was prepared. The 24 h culture of bacteria on the TSB agar plate was harvested and suspended in 5 mL of NaCl solution (0.5 McFarland). Next, the IL was added to a concentration of ½ MIC. Additionally, MgO NPs and SiO_2_ NPs were added in concentrations of 500 mg L^−1^ and 1000 mg L^−1^, respectively. The resulting suspensions were then mixed gently and were left for 1 h without mixing at 25 °C. After the incubation, the suspension was gently mixed and 10 μL was put on a clear glass slide and air-dried. The slides were next fixed over a gas flame and stained with a solution of acridine orange (5 mg, 0.1 L^−1^, pH 7.4) for 1 min. After staining, the bacteria appeared red and the mineral phases appeared green. The slides were examined under the epifluorescence microscope using a blue filter for excitation.

### 2.9. Respirometric analysis

The growth of bacteria in the presence of the studied NPs and ILs was measured using the respirometric system. A MicroOxymax (Columbus Instruments, Columbus, OH, USA) respirometer was used to measure the amount of CO_2_ formed by the microbial activity of the bacteria. The respirometry system was chosen as more convenient in opaque samples with suspension of nanoparticles. Additionally, it is possible to distinguish developing bacterial culture from one in which the bacteria do not develop but are still alive producing CO_2_. It is important when the cytostatic (instead of bactericidal) effect can occur. The test was conducted as follows: 10 mL of the sterile medium (TSB) was placed in a 100 mL sterile glass bottle (Simax). The experiments comprised the following: (a) a control culture without the studied compounds, (b) a culture with NPs at concentrations of 500 or 1000 mg L^−1^ for MgO NPs or SiO_2_ NPs, respectively, (c) cultures with imidazolium ILs at concentrations of ½ MIC, and (d) cultures with NPs and ILs at concentrations of ½ MIC. Then, 0.5 mL of bacterial suspension (approximately 10^8^ colony-forming units (cfu) mL^−1^ in 0.9% NaCl) was added and the bottle was connected to a respirometric system. The cultures were stirred (120 rpm) at 25 °C for 50 h. The amount of CO_2_ produced was measured automatically every 2.5 h.

### 2.10. Statistical analyses

The data were analysed for significant mean differences using a one-way analysis of variance (ANOVA) at *p* < 0.05. *Post hoc* tests for pairwise differences and the identification of homogeneous subgroups were conducted using Tukey's honestly significant difference procedure. The analyses of variance were computed with Statistica 13.1 software (StatSoft, Tulsa, OK, USA).

## 3. Results

### 3.1. Characteristic of nanoparticles

**Fig. 1:**
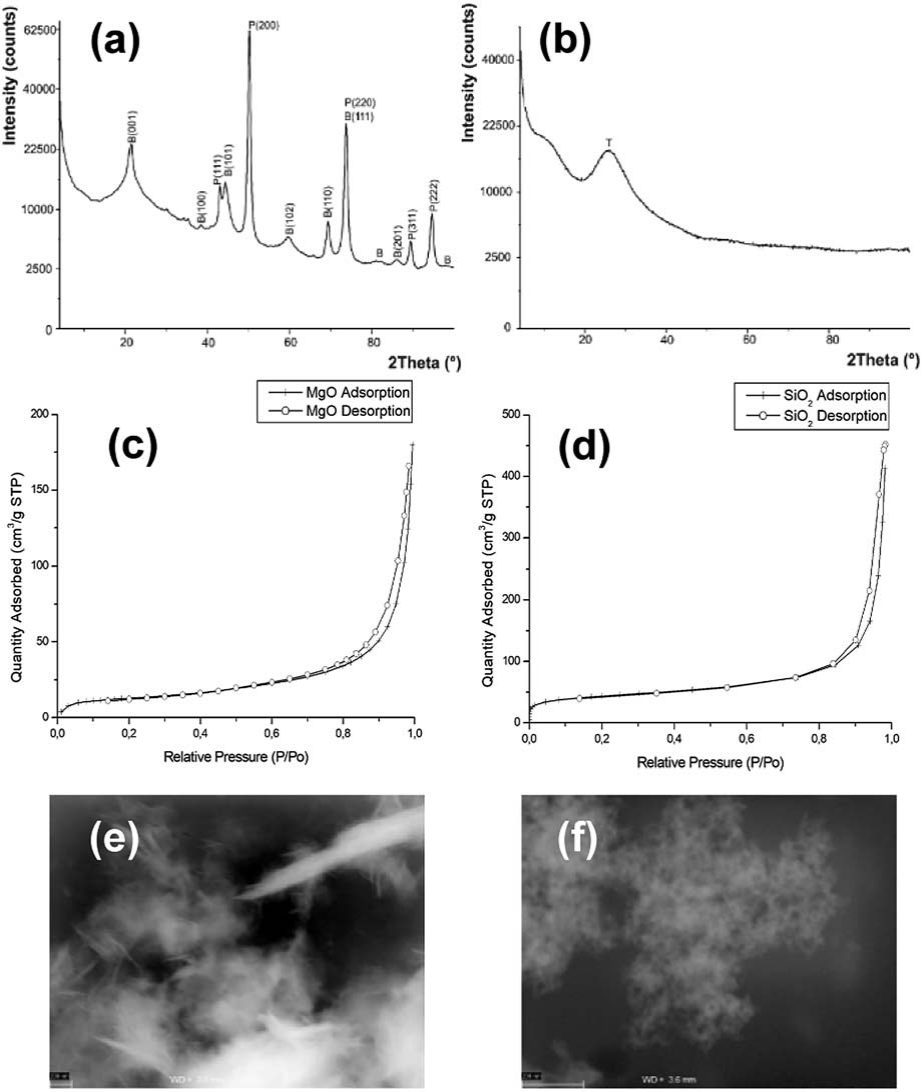
The diffraction pattern of MgO and SiO_2_ nanoparticles: (a) B – reflections from the brucite, P – reflections from the periclase. (b) T – reflections from the tridymite. The BET sorption isotherms: (c) MgO nanoparticles, (d) SiO_2_ nanoparticles. STEM of nanoparticles: (e) MgO nanoparticles, (f) SiO_2_ nanoparticles. Bar – 200 nm.

The XRD analysis of MgO nanoparticle samples showed that the samples contained periclase (MgO) and brucite [Mg(OH)_2_] (Fig. 1a). The SiO_2_ nanoparticle sample consisted of tridymite (Fig. 1b), but the crystal size prevented accurate confirmation of the phase composition. Moreover, the crystal sizes of SiO_2_ nanoparticles were too small to calculate using the Scherrer formula. It was examined using the STEM method, which showed that the size was <10 nm (Fig. 1 e and f). The SiO_2_ nanoparticles were probably at the limit of crystallinity. The size of the MgO nanoparticles was approximately 25 nm for periclase. The crystallite size of brucite was approximately 16 nm in a direction perpendicular to the lattice plane (001) and 27 nm in a direction perpendicular to the lattice plane (100). The size of the corresponding nanoparticles was provided by the manufacturer. The XRD analysis provided the evidence that the MgO nanoparticles were accompanied by magnesium hydroxide, despite the dehydratation process carried out before experiments. However, it is obvious effect of reaction with water, and during the experiments with bacteria in microbial medium, the Mg(OH)_2_ with MgO nanoparticles existed. In case of silica nanoparticles, XRD did not reveal any other mineral phases, however, it was possible that the surface of silica particles under hydrous conditions during microbiological experiments was also partially hydroxylated.

The N_2_ adsorption isotherm of MgO NPs (Fig. 1c) was classified as type IV according to the IUPAC classification, which was characteristic for a dominantly mesoporous material. The hysteresis loop of the MgO adsorption isotherm H3 was attributed in materials with platy particles having slit-shaped pores. The N_2_ adsorption isotherm of SiO_2_ NPs (Fig. 1d) could not be ascribed to the one type of isotherm from the IUPAC classification. It was a mix of type I/IV. The presence of micropores and mesopores or macropores was evidenced by pore size distribution curves and the presence of a hysteresis loop found in other mesoporous ordered silicas. Bimodal distribution of the pore size indicated that two types of pores were present in the sample micropores, which created over one-third of the specific surface area of mesopores and macropores. The BET surface area was 46.65 ± 0.30 m^2^ g^−1^ and 154.89 ± 1.55 m^2^ g^−1^ for MgO NPs and SiO_2_ NPs, respectively. Additional data from BET and PZC analyses are presented in ESI (Table S2†). The PZCs of MgO and SiO_2_ NPs were 11.5 and 3.5, respectively. Thus, we concluded that just below neutral pH (7), the net surface charge for MgO NPs was positive and for silica NPs it was negative.

### 3.2. Toxic effects

**Fig. 2:**
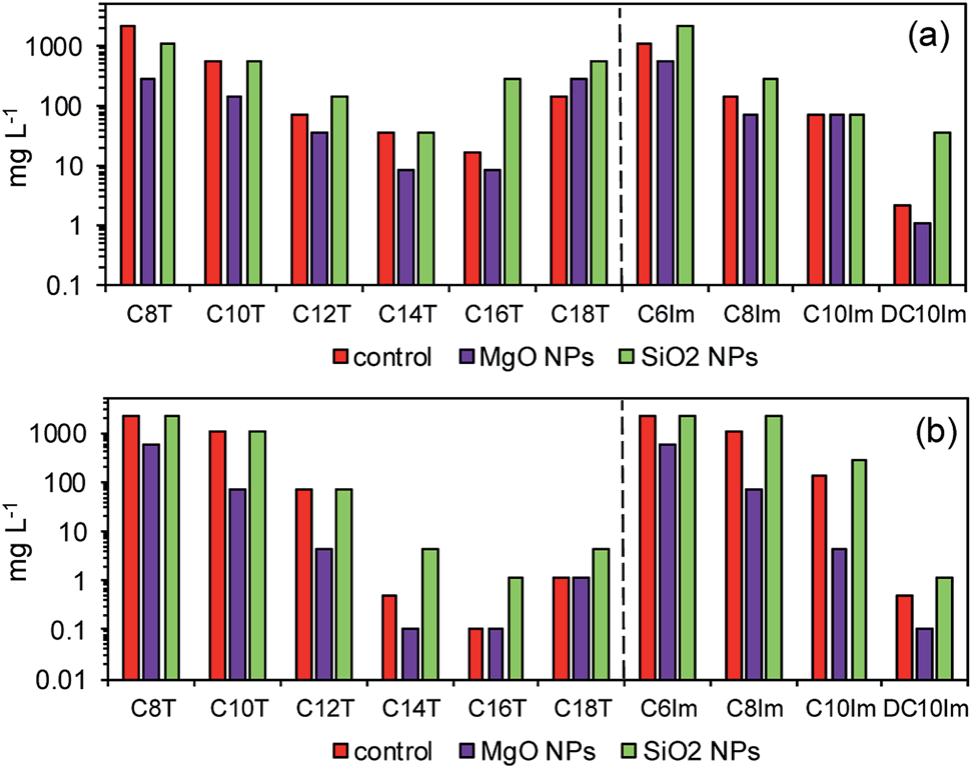
The minimum inhibitory concentration (MIC) of pure solutions of ionic liquids (control) and at approximately ½ MIC of MgO and SiO_2_ nanoparticles for *E. coli* (a) and *B. cereus* (b).

Theophylline-based ionic liquids (TILs) were designated as C8T–C18T depending on the alkyl chain. Similarly, the imidazolium ionic liquids (ImILs) were designated as C6Im, C8Im, C10Im, and DC10Im. The structure of these ILs are shown in Table S1 (ESI†). The toxicity of the studied ILs are presented in Fig. 2 (red bars – control). The toxicity of TILs and ImILs correlated with the alkyl chain length. For *E. coli*, the most toxic were C16T and DC10Im, and the MICs were 17 mg L^−1^ and 2.1 mg L^−1^, respectively. For *B. cereus*, the results were the same; however, the values were lower. The MIC for C16T was 0.1 mg L^−1^ and the MIC for DC10Im was 0.5 mg L^−1^. The toxicity of pure MgO NPs and SiO_2_ NPs were approximately 2000 mg L^−1^ or higher for both *E. coli* and *B. cereus* (Table 1, control). After collecting MIC data for the pure studied compounds, the next experiments were conducted in order to assess the changes of bacterial sensitivity to ILs at ½ MIC of NPs (Fig. 2). In these experiments, the ½ MICs of MgO NPs and SiO_2_ NPs were set as 500 mg L^−1^ and 1000 mg L^−1^, respectively, independent of the bacterial strains.

**Table 1:** The minimum inhibitory concentration (MIC) of nanoparticles at ½ MIC of ionic liquids

MIC [mg L^−1^]	*E. coli*		*B. cereus*	
	MgO NPs	SiO_2_ NPs	MgO NPs	SiO_2_ NPs
Control	2200	>2200	>2200	>2200
C8T	1100	>2200	550	>2200
C10T	550	>2200	280	>2200
C12T	550	>2200	280	>2200
C14T	140	>2200	2200	>2200
C16T	140	>2200	2200	>2200
C18T	280	>2200	2200	>2200
C6Im	280	>2200	2200	>2200
C8Im	70	>2200	1100	>2200
C10Im	280	>2200	1100	>2200
DC10Im	2200	>2200	550	>2200

It can be seen that the addition of MgO NPs caused the decrease of MICs of both theophylline-based and imidazolium-based ILs. Notably, the most important differences occurred in studies of Gram positive bacteria. For example, the MIC for C10Im in control cultures was 140 mg L^−1^, while the MIC for the same IL with MgO NPs decreased to 4.5 mg L^−1^. In one case, the observed result was different. The MIC of C18T for *E. coli* was lower in the control than with MgO NPs. The opposite results were obtained in the evaluation of MICs in the presence of silica NPs. The addition of silica NPs caused either an increase in the MIC of ILs or the measured MIC was the same as the control. Furthermore, the reverse experiment was performed to check whether the MIC of nanoparticles changed in the presence of ½ MIC of ILs (Table 1). The results confirmed the previous conclusion, indicating the synergistic action of ILs and MgO NPs. In the case of MgO NPs, the MIC in relation to *E. coli* decreased from 2200 mg L^−1^ in the control to even 140 mg L^−1^ in the presence of C14T and C16T. The smallest inhibitory concentration was noted with C8Im. A similar, though less distinct relationship, was found for *B. cereus*. The presence of ILs usually caused a decrease of the MIC of MgO NPs. However, the MIC of silica nanoparticles did not change in both bacterial cultures.

### 3.3. Activity of bacteria

**Fig. 3:**
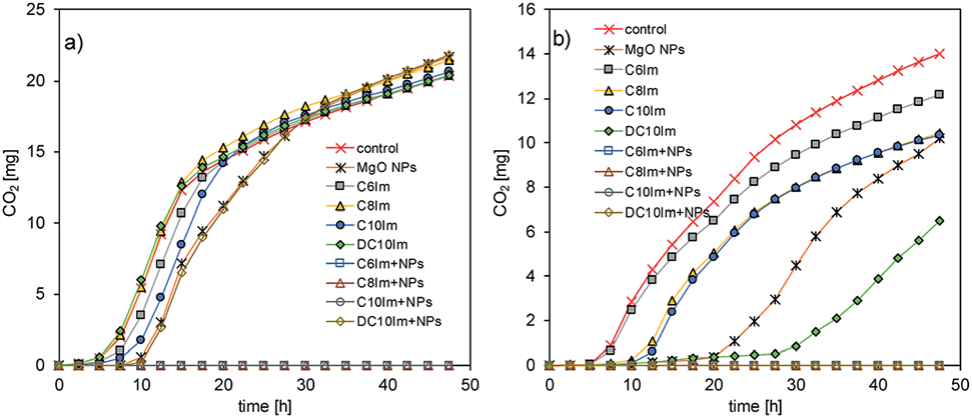
Respirometric analysis of the cultures of *E. coli* (a) and *B. cereus* (b) in the presence of ½ MIC of MgO nanoparticles and imidazolium-based ILs.

The MIC assessment indicated the possibility of a synergistic mode of action of MgO NPs and ILs, and the antagonistic action of silica NPs and ILs. In the next experiments, the activity of bacteria in the presence of ½ MIC imidazolium-based ILs and NPs in below toxic concentrations was examined. The imidazolium-based ILs were chosen from a practical point of view. The studied assemblage of ImILs comprised only four compounds, which differed markedly in their toxicities, from poorly toxic C6Im to highly toxic DC10Im. First, respirometric analyses were conducted (Fig. 3). The addition of MgO NPs together with IL, for both types of bacteria, caused complete inhibition of CO_2_ production, which indicated the inhibition of bacterial activity or even cell death. In contrast, separate addition of MgO NPs or IL did not cause the death of cells, but only affected the activity by extending the lag phase of bacterial cultures. The opposite results were obtained when the SiO_2_ NPs with ILs were used. The CO_2_ production in such cultures of both bacteria did not differ from the control culture, and the resulting respirometric curves overlapped (data not shown).

**Fig. 4:**
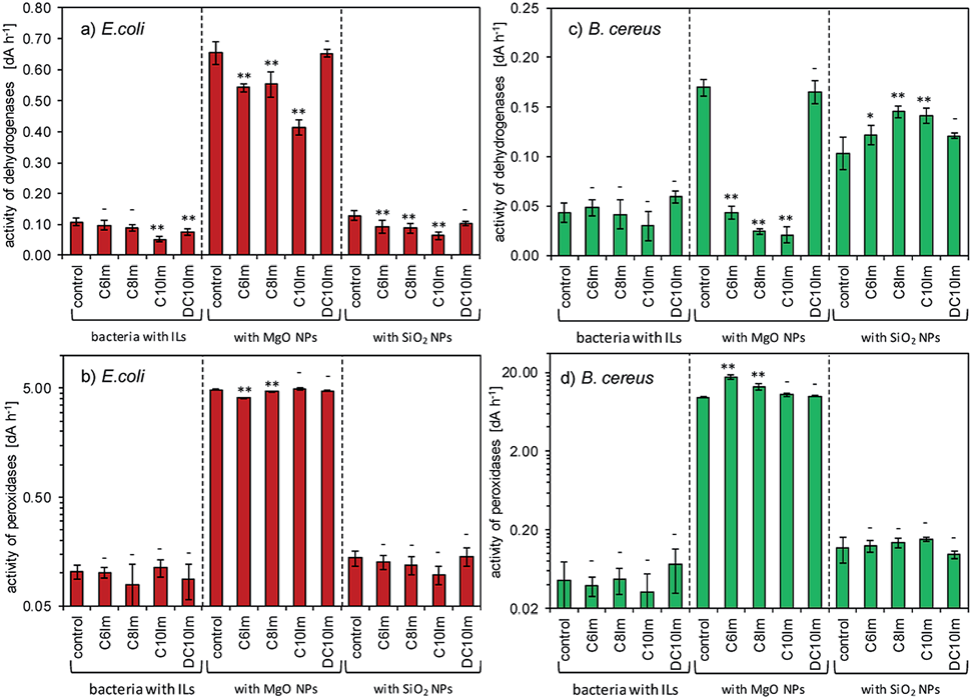
The activities of dehydrogenases and peroxidases in cultures of *E. coli* (a and b) and *B. cereus* (c and d). The standard deviation and statistically significant differences compared to controls are marked: (**) *p* < 0.05, (*) *p* < 0.1; (-) not significantly different.

The above experiment was performed using cultures inoculated and supplemented by NPs and ILs at the same time. However, it was important to determine, whether the dense culture of bacteria, supplemented by NPs and ILs after cultivation, could also become sensitive. It was obvious that the dehydrogenase and peroxidase activities should be changed in such cases. Additionally, the peroxidase activity could be treated as an indirect marker of metabolic disturbances especially involved with oxidative stress. Fig. 4 shows the results of the dehydrogenase and peroxidase activities.

The results showed that the addition of ImILs in the presence of MgO NPs affected the activity of dehydrogenases. It is important to note, however, that the MgO NPs also strongly changed enzymatic activities. Additionally, the observed changes were dependent on the ILs. For example, the most toxic DC10Im did not change the dehydrogenases activity in control cultures with pure MgO NPs, which was found in both bacterial cultures. It should also be emphasized that the presence of NPs with ILs strongly elevated the level of peroxidase activities, such as for C6Im in *B. cereus* cultures. Note that the scales on diagrams (b) and (d) (Fig. 4.) are logarithmic. For silica NPs, no significant changes were found except for the *B. cereus* cultures, where the increased dehydrogenase activity was noted (Fig. 4c).

The above analysed cultures were then examined using scanning electron microscopy. The C6Im cultures were chosen because of the low toxicity of this IL. The control culture with C6Im should not exhibit distinguishable changes in cell morphology using SEM analysis. Additionally, the imidazolium- or quaternary ammonium-based ILs with short alkyl chains usually did not cause cell aggregation or agglomeration, which could interfere with the interaction of NPs with bacteria. Thus, the SEM analysis was performed and provided important results.

### 3.4. Microscopic analysis

**Fig. 5:**
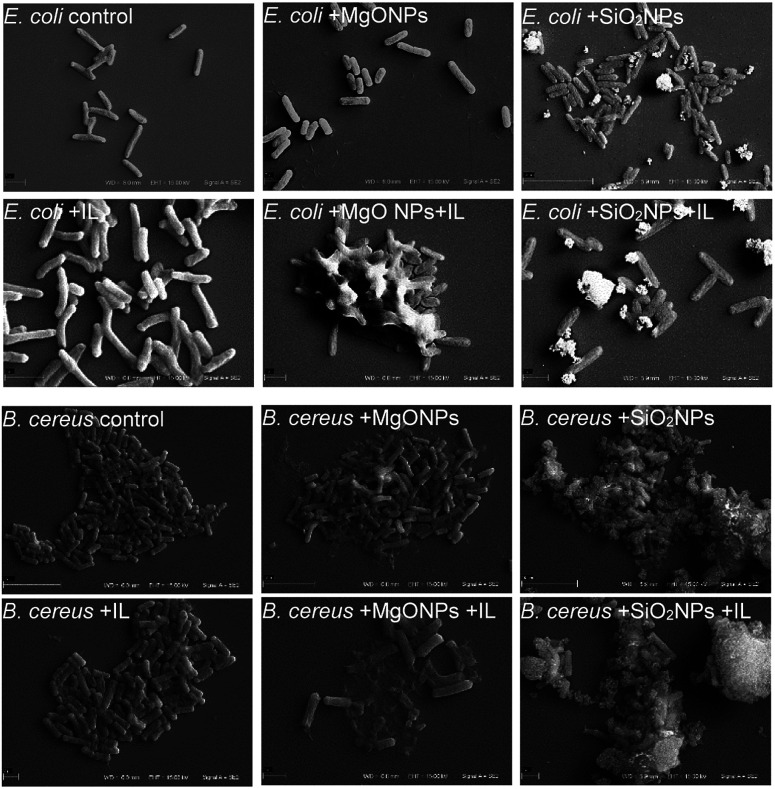
Scanning electron microscope analysis of the bacteria from cultures with C6Im ionic liquid and MgO NPs or SiO_2_ nanoparticles.

The presence of C6Im IL in cultures of both examined bacteria did not cause any changes compared to the control, which was confirmed using SEM analysis (Fig. 5). The C6Im IL was not very toxic so in the wide range of concentrations used, it did not damage the cells. Similarly, the addition of pure MgO NPs to *E. coli* also did not cause cell damage; however, in case of *B. cereus*, some damaged cells were found. Only the addition of C6Im with MgO NPs led to clearly visible cell destruction in both *E. coli* and *B. cereus* cultures. Especially in the case of Gram positive bacteria, significant damage and cell debris were found. Very interesting results were observed in the case of silica NPs. The observed cells of both studied bacteria were intact and no damage was found. Additionally, the bacterial cells seemed to be agglomerated with the nanoparticles, which was not observed in cultures with MgO NPs and IL.

**Fig. 6:**
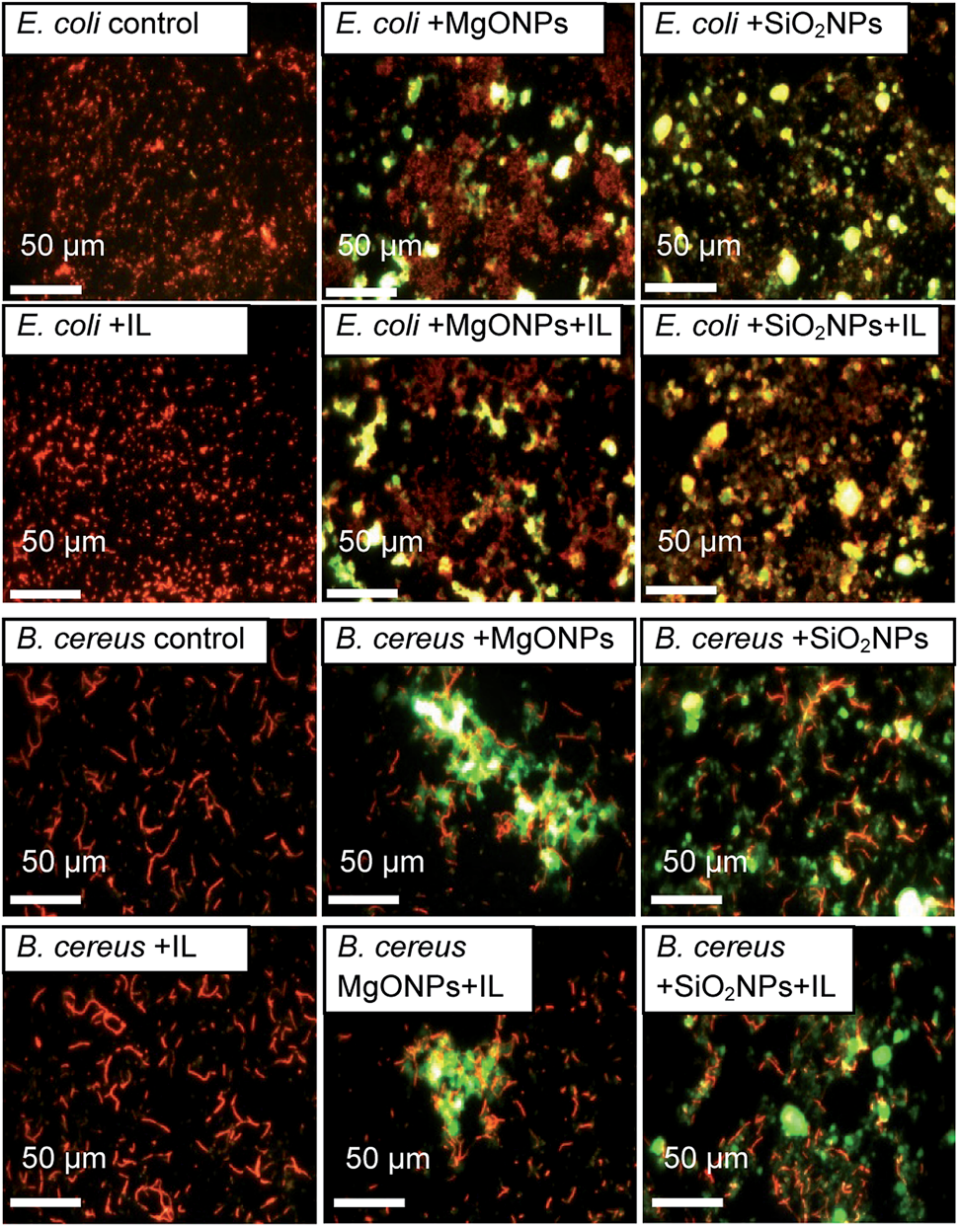
The fluorescence microscopic images of the bacteria from cultures with C6Im ionic liquid (IL) and MgO NPs or SiO_2_ NPs. The bacteria are orange-red, and the mineral phases are yellow-green.

The fluorescence microscopy results also demonstrated that the addition of the IL did not cause cell agglomeration, which was shown previously (Fig. 6). On the contrary, the presence of MgO NPs caused bacterial agglomeration/aggregation, which was clearly visible under a fluorescence microscope (Fig. 6; *E. coli* + MgO NPs). Furthermore, the presence of IL with MgO NPs led to relaxation of bacterial structures. However, this phenomenon was observed only in the cultures of Gram negative bacteria. However, in the case of silica NPs, the presence of NPs with C6Im caused the adsorption of bacterial cells onto the surface of nanoparticle agglomerates. This process was observed in both Gram negative and Gram positive bacteria. The presence of NPs alone did not cause agglomeration of the bacteria or significant adsorption onto the surface.

### 3.5. Interaction of ILs with NPs

**Fig. 7:**
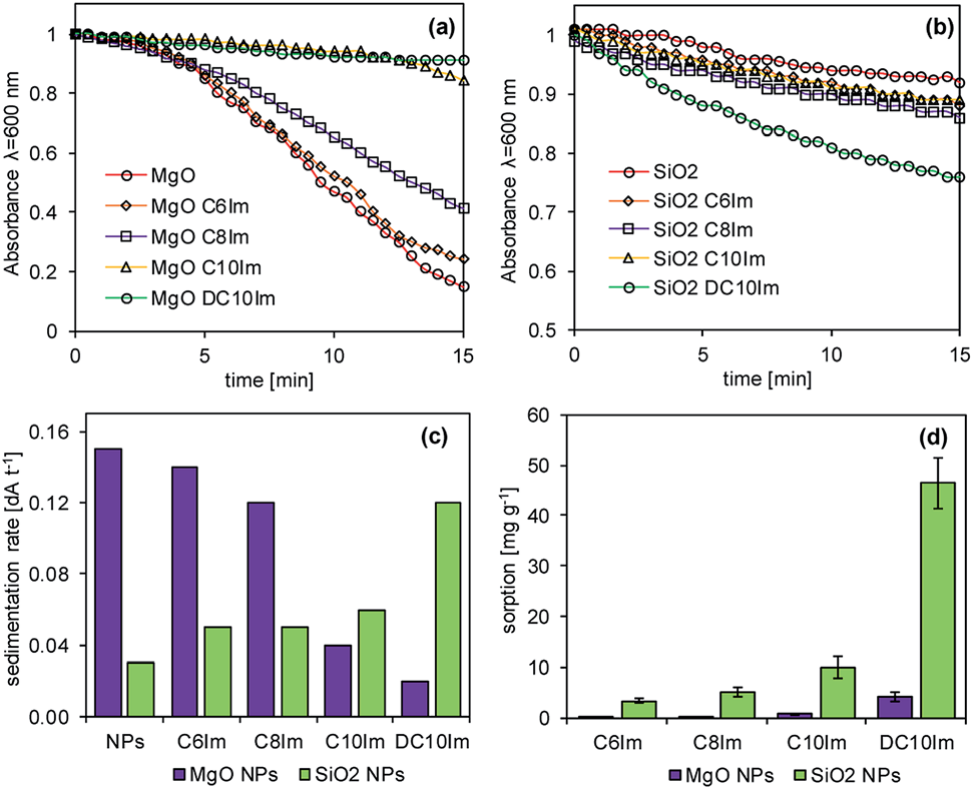
The interaction of ILs with NPs. The sedimentation of MgO NPs (a), SiO_2_ NPs (b), the sedimentation rate (c) in the presence of ionic liquids, and the adsorption of ionic liquids onto the nanoparticles (d). The standard deviation is shown in (d).

Regarding the microbiological experiments described above, it was important to determine whether the ionic liquids interacted with the surface of the NPs. Thus, the adsorption of ILs onto the NPs and rate of NP sedimentation in the presence of ionic liquids were determined (Fig. 7). The results showed that the ionic liquids changed the sedimentation rate of the NPs, and this was dependent on both IL structure and type of NPs. In the case of silica NPs, the sedimentation rate was proportional to the alkyl chain length of the imidazolium of the ILs. In contrast, in the case of MgO NPs, such a relationship was inversely proportional. The adsorption of imidazolium ILs was also noted and was dependent on the chemical structure of the ILs, as well as the type of NPs. The ionic liquids with short alkyl chains (C6Im, C8Im) adsorbed onto MgO NPs very weakly, reaching 0.1 mg g^−1^ and 0.3 mg g^−1^, respectively. Those with C10Im and DC10Im chains were adsorbed more strongly, but the obtained values of adsorption did not exceed 5 mg g^−1^. A similar relationship was noted in the case of adsorption onto SiO_2_ NPs; however the process was more efficient. The adsorption of the samples with C6Im and C8Im chains was 3.3 mg g^−1^ and 5.1 mg g^−1^, respectively. The greatest adsorption was noted with ionic liquid with double decyl substituents in the structure (DC10Im), and the measured adsorption was 46 mg g^−1^.

## 4. Discussion

**Fig. 8:**
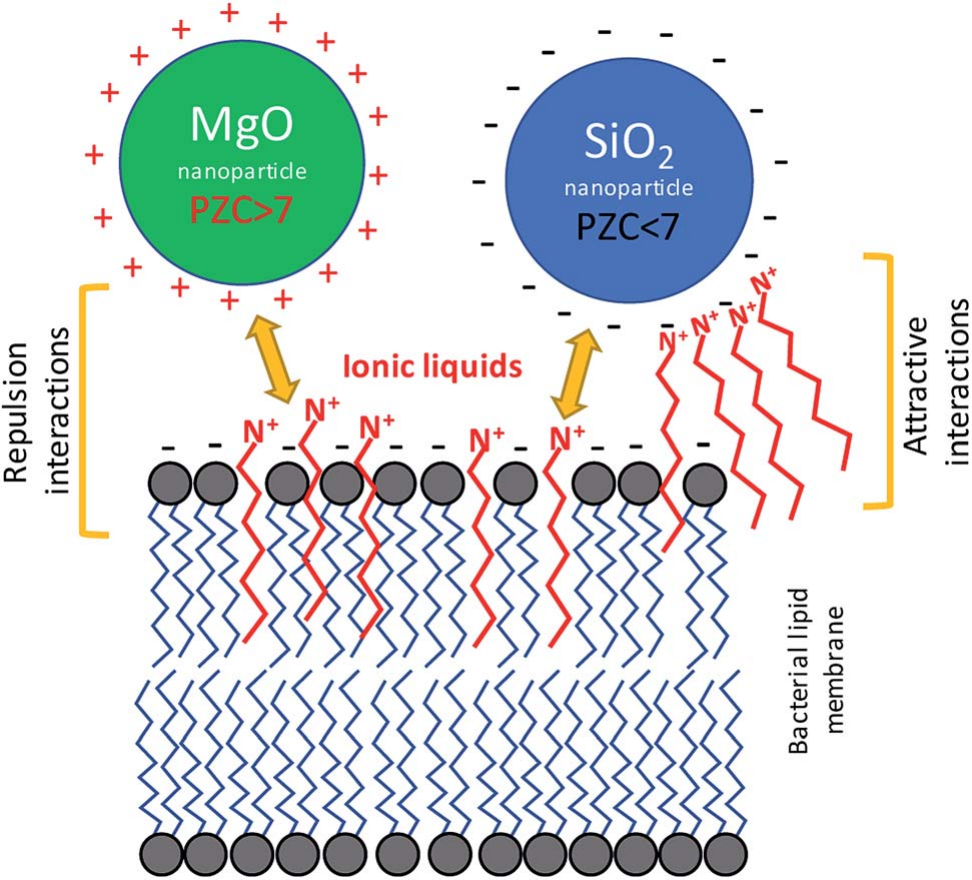
The hypothetical interactions between bacteria, silica or MgO nanoparticles and ionic liquids at neutral pH based on the presented studies. When the cations from dissociated ionic liquids with alkyl chain will adsorb onto negatively charged silica nanoparticles, the attractive interactions with bacteria can occur. However, the positive charge of MgO nanoparticles can hamper the sorption of ionic liquids, but the ILs can intercalate into the bacterial membrane. Thus, the repulsion effects will be observed. On the other hand, the interaction of ILs with membrane can lead to membrane damages and the sensitivity increase to the nanoparticles may potentially reveal.

There is no doubt that the ionic liquids can interact with mineral phases, including nanoparticles, which can lead to change of chemical properties of NPs and ILs as well.^44^ These interactions are not unidirectional. The ILs can interfere with mineral particles changing their surface properties, but the nanomaterials can also modify the physicochemical behaviour of ionic liquids.^45,46^ Despite the interesting aspects of such relationships, the problem of whether the NPs together with ILs can simultaneously interact with bacterial cells remained an open question. The bacterial toxicity of quaternary ammonium ILs and imidazolium ILs was previously reported.^10,26,47^ It is known that the mechanism of toxic action involves mainly the interactions with bacterial cell envelopes.^48^ These ILs, as well as their precursors comprising the alkyl chains, may incorporate into the membrane, which can lead to membrane disruption or increase lipid peroxidation. In the present study, the TILs and probably other quaternary ammonium ILs as well as imidazolium ILs could affect the net surface charge and electrokinetic potential of bacterial cells. Previous studies showed that the electrokinetic potential of bacterial cells can be changed in the presence of amphiphilic ILs.^7^ Usually, the bacterial cells are characterized by having a low charge (PZC), in the range of 2–4. This determines the net surface charge at close to neutral pH.^7,38,39^ However, the interaction with some amphiphilic ILs can change the surface charge dependent on the structure of such compounds and on the membrane composition of the bacteria. This is especially important in relation to Gram negative bacteria, which are enveloped by an outer membrane comprised of lipopolysaccharides. As in the case of these bacteria, the ILs can interact with the surface of the nanoparticles dependent on the available surface area and surface charge of the nanoparticles used for the NPs synthesis.^49^ Therefore, on the one hand the amphiphilic character of ILs allows them to interact with bacterial membranes; on the other hand, the ionic character of ILs can affect the surface of the NPs. Thus, these types of interactions could affect the sensitivity of the bacteria to nanostructured materials. Indeed, our obtained results indicated that the bacterial susceptibility to NPs was affected by ILs (Fig. 8). It should be emphasized that the presence of NPs and ILs at concentrations below the MIC, which were combined in bacterial cultures, can cause strong responses from the bacteria, including having lethal effects. However, distinct differences were observed. The MgO NPs caused the aggregation of bacteria, but in the presence of the ILs this aggregation was smaller. In contrast, the SiO_2_ NPs strongly adsorbed the bacteria in the presence of ILs. Furthermore, the sensitivity of bacteria to MgO NPs rapidly increased when the culture contained the ILs even at concentrations below the MIC. In the case of silica NPs, the effect was the opposite, and the presence of SiO_2_ caused decreases of bacterial sensitivity to the ILs. A similar inverse relationship was also noted in the case of the interaction of ILs with NPs in abiotic conditions.

### 4.1. Interactions in MgO NPs–ILs–bacteria systems

The aggregation of bacteria in the presence of MgO NPs mainly involved the Mg^2+^ dissociating from particles in water suspensions. The bacterial adhesion as well as the bacterial aggregation is controlled by a balance between two additive forces: attractive and universal Lifshitz–van der Waals forces, and repulsive electrical double-layer forces, as described by the Derjaguin–Landau–Verwey–Overbeek (DLVO) theory of lyophobic colloid stability.^50,51^ The electrical double layer originates from the negative charge on the bacterial surface and the negative charge that naturally occurs at most solid surfaces. Neutralization of the electrical double layer can be achieved through electrostatic interaction with cations. In the presence of metallic cations, the anionic groups of the outer membrane of Gram negative bacteria are electrically neutralized. In the case of Gram positive bacteria, the charge neutralisation can concern the bacterial capsule, which is also usually negatively charged at neutral pH. Divalent cations (Mg^2+^ and Ca^2+^) are known to be crucial not only for the neutralization of the electrical double layer between the cell and substratum, but also for the integrity of the bacterial outer membrane.^52^ The presence of ILs additionally disturbs these interactions, due to the additional positive charge, and due to intercalation onto the lipid membrane or bacterial capsule. The PZC of MgO NPs was relatively high, and at neutral pH, the net surface charge was positive, thus the adhesion of bacterial cells with intercalated ILs was hampered. Indeed, the adsorption of bacteria onto the surface of MgO aggregates was not observed. However, the MgO NPs are usually characterised by high surface catalytic activity,^53^ which can generate reactive oxygen species (ROS) that could more easily react with the bacterial cells that were treated by ILs. The results of enzymatic activity measurements suggested such a mechanism. Based on the results of sorption of ImILs onto the MgO NPs and the sedimentation rate, it can be stated that the ImILs caused better dispersion, hindering the aggregate creation. Additionally, the ImILs did not undergo strong adsorption onto the MgO NPs.

### 4.2. Interactions in SiO_2_ NPs–ILs–bacteria systems

In the case of silica NPs, the main mechanism controlling the interactions with bacteria and ILs probably involved adsorption processes. The SiO_2_ NPs adsorbed much stronger than the ionic liquids, and were dependent on the alkyl chain length. This process could have caused the two main effects that were noted in our study. First, the sensitivity of bacteria to ILs was significantly decreased, which was especially evident in the case of more toxic ionic liquids with longer aliphatic chains; and second, the presence of both SiO_2_ NPs and C6Im IL could have caused the adsorption of bacteria onto surface of silica NPs aggregates in water suspensions. Both phenomena could be easily explained by adsorption processes. The PZC of silica NPs is quite low, and under neutral pH, the net surface charge is negative. Adsorption of cations from ILs could lead to changes of surface charge, which may result in the greater adsorption of bacterial cells. Additionally, the cationic component of the ILs comprises the alkyl chains; thus, the interaction of C6Im with the bacterial membrane can also lead to the same effect because such cells should be more easily adsorbed onto the silica aggregates. The silica NPs in suspension with the ILs underwent agglomeration or aggregation dependent on the alkyl chain length, in the IL structure. Thus, the formation of agglomerates/aggregates can disturb the nano-character of SiO_2_ NPs. The interaction of bacteria with silica NPs did not have any toxic effects. Such effects were also not observed in the presence of ILs and SiO_2_ NPs.

## 5. Conclusions

Ionic liquids comprised of alkyl chains can exhibit amphiphilic properties, and therefore, can interact with bacterial membranes, affecting the electrochemical properties of bacterial cells. Based on our results, it can be stated that the same properties govern the interaction in bacteria/nanoparticles/ionic liquids systems. The high PZC of MgO NPs probably caused the poor adsorption of ionic liquid cations with aliphatic chain. On the contrary, the low PZC of silica NPs promoted higher ILs adsorption. The amphiphilic character of the ILs may lead to interactions with bacterial membranes. Thus, the main effects of interactions of bacteria with ILs and MgO NPs likely involved increased cell membrane sensitivity to catalytic activity by the MgO nanoparticle effects. By increasing the bacterial sensitivity to ILs in the presence of MgO NPs higher peroxidase activity than that of control cultures was observed. In the case of silica NPs, the main effect was a decreased sensitivity to ILs in the presence of NPs. Thus, the stronger adsorption of ILs onto the surface of silica NPs may have caused the decrease of concentration of ILs in bacterial cultures. Additionally, the sorption of ILs onto the silica NPs affected the net surface charge, which led to a greater bacterial affinity for silica aggregates in water suspensions. In conclusion, the two opposed effects can be observed. The increase of MgO NPs toxicity in presence of ILs, and the decrease of ILs toxicity in presence of silica NPs.

## Conflicts of interest

There are no conflicts to declare.

## Supplementary Material

RA-009-C9RA05110D-s001
