# Supplementary material for: Ionic liquids strongly affect the interaction of bacteria with magnesium oxide and silica nanoparticles
Source: RSC Adv. 2019 Sep 12;9(49):28724–34. doi: 10.1039/c9ra05110d (PMC9071200; doi:10.1039/c9ra05110d)
Supplement: RA-009-C9RA05110D-s001 [file RA-009-C9RA05110D-s001.pdf]

Electronic Supplementary Material (ESI) for RSC Advances.
This journal is © The Royal Society of Chemistry 2019

Supplementary materials

Table S1. The theophylline-based ILs and imidazolium-based ILs used in the studies.

Compound	Name<br>Molecular weight [g/mol]	Structure
C8T	Octyltrimethylammonium theophyllinate<br>MW: 351.49	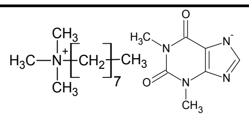
C10T	Decyltrimethylammonium theophyllinate<br>MW: 379.54	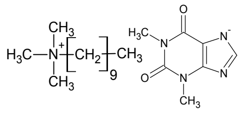
C12T	Dodecyltrimethylammonium theophyllinate<br>MW: 407.59	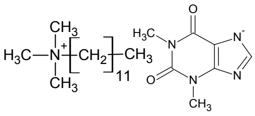
C14T	Tetradecyltrimethylammonium theophyllinate<br>MW: 435.65	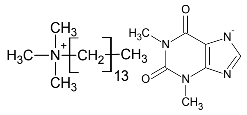
C16T	Hexadecyltrimethylammonium theophyllinate<br>MW: 463.70	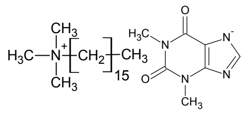
C18T	Octadecyltrimethylammonium theophyllinate<br>MW: 491.75	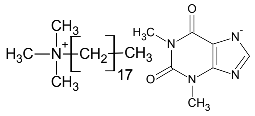
C6Im	1-hexyl-3-methylimidazolium chloride<br>MW: 202.72	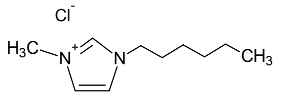
C8Im	1-methyl-3-octylimidazolium chloride<br>MW: 230.78	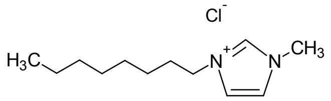
C10Im	1-decyl-3-methylimidazolium chloride<br>MW: 258.83	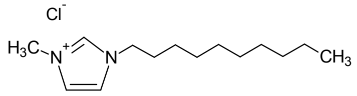
DC10Im	1,3-didecyl-2-methylimidazolium chloride<br>MW: 399.10	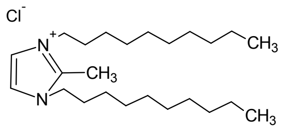

Table S2. BET and PZC analyses of studied nanoparticles

	MgO nanoparticles	SiO_2 nanoparticles
BET Surface Area	46.6530 ± 0.3040 $m^2\ g^{-1}$	154.8858 ± 1.5541 $m^2\ g^{-1}$
Correlation Coefficient	0.9998729	0.9998509
t-Plot Micropore Area	6.4513 $m^2\ g^{-1}$	63.0977 $m^2\ g^{-1}$
t-Plot External Surface Area	40.2017 $m^2\ g^{-1}$	91.7880 $m^2\ g^{-1}$
Point of Zero Charge	11.5	3.5
